# Treatments of palpebral congenital melanocytic nevus: a systematic review

**DOI:** 10.1590/acb384823

**Published:** 2023-12-01

**Authors:** Cristina Pires Camargo, Marita Saliba, Elio Assaad Saad, Milanie Milan, José Mauricio Caldera

**Affiliations:** 1 Universidade de São Paulo – School of Medicine – Laboratory of Microsurgery and Plastic Surgery – São Paulo (São Paulo) – Brazil.; 2 University of Balamand – Faculty of Medicine – Beirut – Lebanon.

**Keywords:** Nevus, Pigmented, Eyelid Neoplasms

## Abstract

**Purpose:**

Palpebral congenital melanocytic nevi (PCMN) is a rare congenital skin lesion affecting the eyelids that can lead to cosmetic and psychological concerns and potential health risks such as malignancy. Several authors have analyzed therapeutical strategies to treat PCMN. However, there was no consensus in the literature. This systematic review aimed to evaluate the effectiveness, safety, and success of treatments of PCMN.

**Methods:**

We conducted a systematic review following PRISMA guidelines from October 2022 to April 2023. We included all types of study designs that described or compared PCMN treatments and interventions, as well as histology, recurrence, adverse events, patient satisfaction, and malignant transformation. The search strategy was based on specific search words through the following databases: PubMed, Embase, Latin American and Caribbean Health Sciences Literature (Lilacs), Web of Science, and Scopus. Ongoing studies and gray literature studies were included.

**Results:**

We analyzed 25 case reports with 148 participants. The effectiveness, success, and satisfaction with various treatments for PCMN depend on the specific treatment method and the individual patient’s case.

**Conclusions:**

Most of the studies showed that surgical procedures (exeresis) are able to treat PCMN in the eyelid. The variability in outcomes emphasizes the importance of further research to better understand the most effective and safe approaches for treating congenital melanocytic nevi.

## Introduction

Congenital melanocytic nevi (CMN) are nevi that are usually present at birth or within a few weeks and have prevalence of 0.5 to 31.7%^1^, with the majority measuring less than 1.5 mm in diameter^2^^,^ 3 and 1 in 6,900 infants having CMNs larger than 4.1 mm in diameter^4^. A particular presentation is the divided nevus in the palpebra, which is also known as split ocular nevus, kissing nevus or panda nevus. The first description of the kissing nevus, a rare congenital nevus, was published by Fuchs in 1919^5^.

One of the kissing nevus etiology hypotheses is a single nevus development during early embryonic life when the lids are still fused (9^th^ to 24^th^ week gestation). Eyelids separation usually occurs after the 24^th^ week and might coincide with the division of the nevi, having one on the lower and one on the upper lid^6^.

These lesions can be aesthetically disfiguring, causing psychological distress and functional problems such as ptosis, ectropion, epiphora and amblyopia due to exophytic growth^7^ and cancer degeneration^8^.

The lifetime risk of malignant degeneration in small congenital nevi is not clearly established, but large cutaneous melanocytic nevi (> 4 cm) can give rise to melanoma (risk being 4.6% during a 30-year period)^9^. Since the risk of malignancy can’t be ruled out, surgeons advise its removal^7^.

Before discovering the metastatic transformation risk, treatment included observation and delayed surgery, but has shifted nowadays to a more aggressive surgical approach as the first 3–5 years of life constitute the timeframe with the highest number of prepubertal melanoma cases^10^.

Therefore, the goal of treatment is palpebral congenital melanocytic nevi (PCMN) exeresis, improving cosmetic outcomes while minimizing functional impairment and malignancy^11^.

There are several therapeutical strategies to PCMN exeresis. Usually, treatment is performed by surgical excision and full-thickness skin grafts or local flaps, laser therapy, cryotherapy and dermabrasion^6^^,^ 7. However, there was no consensus on the best therapy to treat PCMN.

This systematic review aimed to analyze PCMN treatment, recurrence rate and adverse events.

## Methods

This study is a systematic review based on the PRISMA guidelines. We conducted this study from October 2022 to April 2023. We analyzed all types of study designs describing or comparing PCMN treatments and interventions, as well as histology, recurrence, adverse events, patients’ satisfaction and malignant transformation. We considered trials in which participants are from all genders and of any age who presented with PCMN.

The search strategy was based on the following search words: (melanocytic OR nevus, pigmented OR pigmented nevi OR pigmented moles, melanocytic OR melanocytic nevus OR kissing nevus) AND (eyelid OR palpebra) AND (congenital).

The following databases were used for the search strategy: PubMed, Embase, Latin American and Caribbean Health Sciences Literature (Lilacs), Web of Science, and Scopus; and ongoing studies: ISRCTN registry, ClinicalTrials.gov, Australian New Zealand Clinical Trials Registry, World Health Organization International Clinical Trials Registry Platform, and EU Clinical Trials Register.

We also analyzed gray literature studies and syllabi of the most relevant congresses, the American Dermatology Association Meeting and American Society of Plastic Surgery Meetings, for the last two years.

The primary endpoint of the systematic review was the treatment success rate of PCMN. The second endpoints were recurrence rate, PCMN histology, patients’ satisfaction, adverse events, and malignant degeneration.

After a thorough searching process, we uploaded the files to EndNote Software (Endnote, Clarivate, United States of America), identified and removed the duplicates, to then upload them to the Rayyan platform.

The first selection process was conducted independently by two authors (MS, MM), and all records were analyzed based on title and abstract. The second selection process was conducted independently by three authors (MS, MM, EAS) and was based on full-article assessment. Any disagreement was resolved by a fourth author (CPC).

### Data extraction

In an Excel spreadsheet, we collected the following data: study-ID, study design, country, age, gender, number of participants, PCMN treatment, recurrence rate, adverse events, malignant degeneration.

## Results

**Figure 1:**
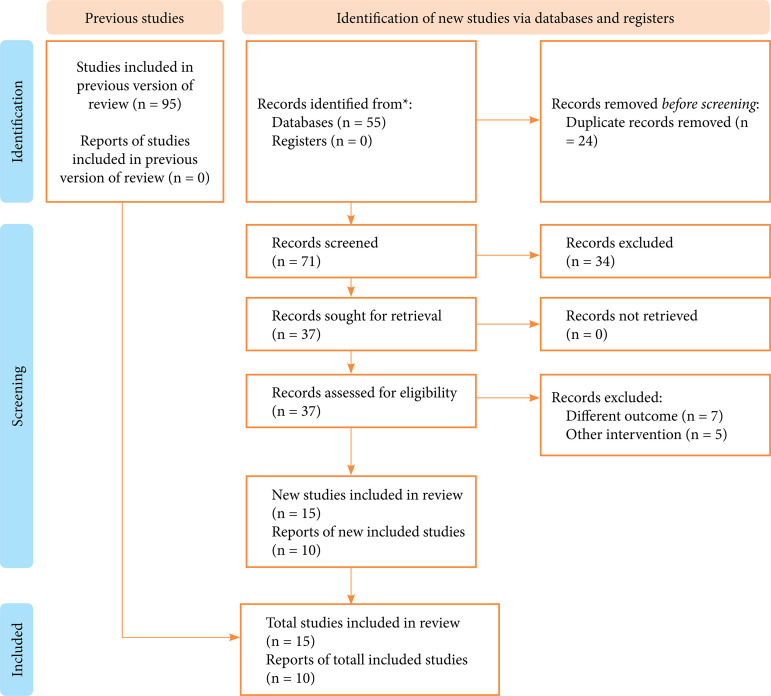
Flow diagram.

Source: Page et al.^12^.

We retrieved 95 articles, removed 24 duplicates, and selected 25 articles after the first and second selection process (Fig. 1).

We selected 25 articles with 148 participants in total, ages ranging from 2 months to 73 years old, with 60.38% being females and 39.62% being males. The dimensions of the nevus ranged from 5 mm to higher than 60 cm.

A number of studies were conducted in China (n = 2), France (n = 1), Nepal (n = 1), Ireland (n = 1), United States of America (n = 4), Turkey (n = 2), United Kingdom (n = 1), Japan (n = 1), India (n = 2), and Italy (n = 1). The studies were case reports (n = 23), case series (n = 1), and retrospective study (n = 1).

## Palpebral congenital melanocytic nevi treatment

A number of treatment options for kissing nevi were developed throughout the years and have been reported in the literature.

### Lasers: treatment success rate of palpebral congenital melanocytic nevi

A study conducted by Gu et al.^7^ analyzed the effect of carbon dioxide laser and erbium:yttrium aluminum garnet laser to treat congenital melanocytic nevi of the periorbital and eyelid regions (five sessions per nevis). Clinical efficacy was evaluated at least six months after treatment by visual assessment and L*a*b* color space, measuring color value of the lesion and normal skin. The degree of CMN lesion improvement was poor in 25% of the patients, fair in 20%, good in 25%, excellent in 20%, and clear in 10% of the patients, all based on visual evaluation.

Two other papers analyzed the efficacy of the CO2 and Er:YAG and association of these lasers (CO2 and Er:YAG) to treat CMN^3^^,^ 13 ^,^ 14. The Er:YAG laser was more accurate in terms of ablation, resulting in short reepithelialization and erythema duration^15^^–^ 17. Two patients demonstrated shrinkage in lesion size after laser therapy^6^. In around 50% of the patients, the outcome was either fair or poor, despite multiple treatment sessions, and surgical excision was indicated.

A case report by Zeng^18^ presented a 25-year-old female patient with PCMN (1.2 × 1.5 cm in size in the lower eyelid and 0.8 × 1 cm of the upper one, with eyelashes and nasolacrimal canal not being involved) of the right eyelid since birth. Thermal damaging of the lower dermis was avoided. Good cosmetic results were obtained after CO2 laser treatment of CMN with no functional impairment, except for minimal repigmentation of upper lid, superficial scar of the lower lid and hypopigmentation on both wounds at the five-month follow-up.

Another case report published by Abrusci and Benzecry^19^ showed a 16-year-old female patient with medium right lower eyelid congenital nevus treated with both CO2 and Er:YAG laser simultaneously. During the session, the nevus was treated with one pass of the 6W power, 4-mm spot Sharplan 40C CO2 laser on continuous wave mode (CWCO2), then with three passes of the 17W power, 6-mm spot SilkTouch on superpulsed mode (SPCO2). Finally, the lesion and a 3-mm radial margin were treated with three passes of the erbium:yttrium-aluminum-garnet (Erbium-YAG) Derm 20 on 15W power, 4-mm spot, and 50% overlap. Within 10 days, total reepithelialization was attained, and no complications were observed during a two-year follow-up. The only finding observed was a satisfactory 3 × 7-mm post-inflammatory lightly hyperpigmented area, not corresponding to melanocytic nevus as per dermatoscopic assessment.

Few authors described better outcome by early treatment with laser in infancy^20^^,^ 21, but this and other studies could not conclude the same^22^.

Therefore, for smaller superficial and well-circumscribed nevi, CO2 laser is recommended.

Moreover, Ponomarev et al.^23^ reported the case of a 55-year-old male and a 30-year-old female patient with CMN on the forehead and lower eyelid. Selective photothermolysis is one of the main advantages of laser therapy when compared to traditional methods of pigmented skin removal, as it thermally and selectively necrotizes melanosomes without causing any thermal damage to the neighboring tissues. The two cases included in this study were treated with 511 and 578 nm dual wavelength copper vapor laser (CVL) radiations with a 3:2 pulse mode power ratio. The laser beams were focused on a 1-mm diameter spot which was 1 mm away from consequent light spots. Using the CVL laser at 0.7–0.85 W and 0.3 seconds exposure time, two sessions, one month apart, were completed for the 55-year-old patient. The 30-year-old patient was treated in the same way, but with three laser sessions of one-month interval. The healing time was three weeks, and during a 24-month follow-up, no recurrences were noted.

### Lasers: palpebral congenital melanocytic nevi histology

The studies mentioned did not analyze PCMN histology.

### Lasers: patients’ satisfaction

After short-term and long-term follow-up, all patients reported satisfactory outcomes.

### Lasers: adverse events

While lower recurrence and complications rates were achieved by Q-switched Ruby, alexandrite, and Nd:YAG lasers, results were temporary, as repigmentation occurred in ruby laser treated patients, for example^23^.

Using the dual-wavelength, CVL is reasonable as the 511 and 578 nm wavelengths coincide with the melanin absorption wavelength and maximum oxyhemoglobin absorption wavelength, respectively. Adjacent tissue thermal damage can be minimized with adequate laser pulse duration, which does not exceed the target chromophore unit’s thermal relaxation time^22^ that depends on the size of the chromophore unit: less than 1 μs for isolated melanosomal cells of 7-μm size and 10 ms for cluster sizes of about 100 μm^24^. Vascularization increases by two folds according to optical coherence tomographic data in the CMN lesion^25^, promoting the use of 578-nm yellow CVL radiation to prevent recurrence by heating dysplastic vessels^26^^,^ 27.

### Lasers: malignant degeneration

The studies mentioned did not analyze malignant degeneration.

### Staged mosaic punching excision: treatment success rate of palpebral congenital melanocytic nevi

Cho et al.^28^ reported the case of 15 patients (10 women and five men with the mean age of 26 years old and ranging from 13 to 73 years old), all presenting with congenital divided nevi involving the eyelash-bearing area. They proposed staged laser excision as the treatment modality to minimize functional deformity of the eyelid. Eight lesions were small (< 1.5 cm), involving only the upper and lower lids, and four lesions were medium (1.5 to 19.9 cm), involving the medial or lateral canthus. Only seven of these patients had laser therapy, eight underwent concomitant excision or had a history of previous excision, and one patient underwent skin graft over the medial canthus area. The mean follow-up period was 10 months with no noted recurrence.

The procedure of staged mosaic pattern punching excision using a 10,600-nm CO2 pulsed laser (UltraPulse, wavelength 10 Hz, impulse, pulse duration = 250 ls, Smart PS, UTI) with impulses of 500 mJ avoids the complete obliteration of the nevus in a single stage, but instead removes it gradually, preserving optimal aesthetic structures like eyelid margin and eyelash-bearing area.

### Staged mosaic punching excision: palpebral congenital melanocytic nevi histology

The studies mentioned did not analyze PCMN histology.

### Staged mosaic punching excision: patients’ satisfaction

Cho et al.^28^ analyzed patients’ satisfaction through a questionnaire that was given to patients at the end of treatment and 90 days post-treatment. All patients were satisfied by the functional and cosmetic results. The eyelid ciliary margins continuity was maintained in 13 of the patients, with minimal irregularity along lid margin. In all patients, partial loss of cilia was noted, but 50% of the upper eyelashes were preserved. No patient was presented with significant complications such as hypertrophic scar, corneal erosion, trichiasis or ectropion. Immediately post-procedure, all patients were very satisfied by the functional and cosmetic results. Three months later, 11 of the patients were very satisfied, whereas three were satisfied, as per the questionnaire.

### Staged mosaic punching excision: adverse events

The study mentioned showed minor adverse events, such as pigmentation, in the short-term follow-up period.

### Staged mosaic punching excision: malignant degeneration

The study mentioned did not analyze malignant degeneration.

### Cryotherapy and dermabrasion: treatment success rate of palpebral congenital melanocytic nevi

In a study, Ehlers reported the use of cryotherapy as treatment^29^, and Miller and Becker^30^ and Johnson^31^ reported the use of dermabrasion as treatment for the pediatric population. They presented the case of a newborn female infant who presented with a left 2 × 2.5-cm pigmented divided nevus associated with thickening of the upper and lower eyelids and mild left eye ptosis. The patient underwent, under general anesthesia, dermabrasion of the nevus, which was shown to have eminent junctional activity and pigmentation according to punch biopsy.

### Cryotherapy and dermabrasion: palpebral congenital melanocytic nevi histology

The studies mentioned did not analyze PCMN histology.

### Cryotherapy and dermabrasion: patients’ satisfaction

Ehlers et al.^29^ showed that in a four-week follow-up the nevus size and pigmentation were decreased, but both size and color reverted to the initial presentation. Therefore, nevus excision and replacement with postauricular full-thickness skin grafts were done, and results at month 2 postoperative assessment were satisfactory, with no pigmentation recurrence.

### Cryotherapy and dermabrasion: adverse events

McDonnel and Mayou^32^ reported failure of wound healing of nevus region and regrowth prevention of the periorbital nevus when using dermabrasion, leading to further excision and full-thickness skin grafting.

### Cryotherapy and dermabrasion: malignant degeneration

The studies mentioned did not analyze malignant degeneration.

### Excision, graft and flap: treatment success rate of palpebral congenital melanocytic nevi

A study by Yap and Earley^33^ reported a 4-year-old boy presenting for left congenital periorbital pigmented nevus. The nevus was excised first, then reconstructed using full-thickness post-auricular skin graft. The nevus of the upper eyelid was excised, then followed by surgical excision of the margins of the lids, including excess eyelashes. Histological studies showed that it was a benign compound nevus.

Another female patient who was 25 years old presented for congenital pigmented periorbital nevus that was associated with recent bleeding after minor trauma, with the pigmentation more accentuated along the eyelashes’ line. Lower-lid excision was performed, including eyelashes, and reconstruction using full-thickness post-auricular skin grafts was carried out. The lesion of the upper lid was excised as a wedge. The eyelid margin was diathermied, with the upper lashes preserved for cosmetic purposes^33^.

A 4-year-old presented for right congenital periorbital pigmented nevus. A two-stage excision was performed, and reconstruction using full-thickness post-auricular skin graft was carried out. Wedge excision was required two years later^33^. Finally, a 23-year-old female patient presented congenital pigmented periorbital nevus that affected the right eyelids and infraorbital region. Numerous excisions were performed, also with reconstruction using full-thickness post-auricular skin grafts^33^.

Another case report by Yildirim et al.^34^ presented a 16-year-old boy with continuous pigment lesion that was affecting the upper and lower left eyelid since birth. The lesion was extended vertically to tarsal conjunctiva of both eyelids. In this case, 3-4 mm of normal skin surrounding the nevus was removed, as well as 1-2 mm from the tarsoconjunctival area. The tarsus with tarsal conjunctiva and cilia were excised due to involvement. The lateral canthal ligament’s upper and lower branch was scissored, leaving the actual ligament intact. A U-shaped tarsoconjunctival flap was prepared and sutured into the remnant outer canthal ligament. From the surrounding tissue of the upper eyelid, a musculocutaneous flap was supplied. From adjacent outer skin, a full-thickness skin graft was prepared to reconstruct lower eyelid defect. The conjunctiva was joined with skin graft, and minimal lateral tarsorrhaphy was done in order to reduce interpalpebral width. Surgical outcome was successful within six months post-surgery.

Papadopoulos et al.^35^ presented four patients (three females and one male), aged from 17 to 54, who underwent surgical treatment for congenital nevi. One of the patients had small sized cellular divided nevus of central eyelid without canthal involvement, and both lids underwent treatment at the same stage. The remaining three patients had treatment in two stages, addressing the lower eyelid first, since the authors believed that they could achieve more predictable results regarding eyelid closure. Incomplete excision of lesions was opted by one of the patients in order to preserve eyelashes and lid margin. Full-thickness pentagon excision was used successfully, since it was found to be very useful in the debulking of nevi that extend in the posterior lamella of eyelids, while maintaining good lobe apposition and preserving the line of eyelashes.

Ghosh et al.^36^, on the other hand, presented the case of a 39-year-old female patient with large kissing nevus spanning the central two third portion of the lower and upper left eyelids, with ectropion, ptosis, trichiasis and distichiasis as a result of lid margin thickening and hair follicle dystrophy. Surgical reconstruction was done in a one-stage fashion by splitting the posterior and anterior lamellae and debulking the upper/lower lid lesions. Debulking the anterior lamella was preceded with a full-thickness pentagonal excision of the posterior lamella on the upper lid, as well as a same type of excision of the lower tarsus, making the lower lid more aligned and rigid. End-to-end anastomosis was used to close the upper and lower lids’ posterior lamellar defects, and, while the upper lid’s anterior lamellar defect had a minor bare area that allowed for granulation and secondary intention healing, the lower lid’s anterior lamellar defect required supraclavicular skin grafting.

Rajput et al.^9^ discussed the case of a 40-year-old female who presented with melanocytic nevi involving both eyelids, and underwent surgery in which canthotomy, and cantholysis were done, and lid repair in both the lids was carried out by the direct suturing method. The entire upper eyelid, including the nevus, was excised. On her follow-up visit, skin over the lids was normal and healed, and visual acuity improved.

Another case presented by Bayramiçli et al.^37^ is of a 14-year-old boy presenting with CPN involving upper and lower eyelids, the eyebrow, and part of the malar eminence and the nasal dorsum, undergoing multi-staged surgical treatment.

Staged excision of the lesion and defect reconstruction with expanded forehead flaps and a temporal island flap with hair-bearing skin was planned. Three months later, the patient was operated on for minor revisions. The final stage of the surgical treatment was performed 14 months later. Two and a half years after, no functional deficiency was noted. In this case, the expanded hairless frontal skin provided excellent flap coverage for the upper and lower eyelids and the medial canthal area.

Finally, Desai et al.^38^ presented an 8-year-old male patient with congenital nevocellular nevus that was treated through excision and local advancement flap with a V to Y closure of the defect lateral to lateral canthus and full-thickness skin graft for eyelid reconstruction. The patient had no complications, and results were satisfactory.

### Excision, graft and flap: palpebral congenital melanocytic nevi histology

The studies mentioned did not analyze PCMN histology.

### Excision, graft and flap: patients’ satisfaction

All studies reported good to excellent results noted by the patients.

### Excision, graft and flap: adverse events

Post-operatively, the 25-year-old patient developed ectropion that was corrected using lateral wedge excision^33^.

Due to the close excision in the lacrimal apparatus region, the 23-year-old patient developed epiphora^33^.

Papadopoulos et al.^35^ noted that one of the patients had epiphora with visual field impairment, while the rest were either asymptomatic or presenting with mild symptoms. Amblyopia was absent in all patients, although patients had eyelid malposition related to cellularity of nevi. The eyelids and lacrimal puncti were pushed away from cornea when lesions were involving medial canthal area, causing epiphora.

### Excision, graft and flap: malignant degeneration

The studies mentioned did not analyze malignant degeneration (Table 1).

**Table 1:** Summary of treatment options for palpebral congenital melanocytic nevi in the literature.

Study_id	Study design	Population	Treatment	Endpoints	Results	Adverse events
Gu et al.^7^	Case series	20 participants (14 females, six males)	Carbon dioxide laser (n = 11); Erbium:yttrium aluminum garnet laser (n = 2); Association (n = 7); Two to 21 sessions	Six months after treatment by visual assessment and L*a*b* color space, measuring color value of the lesion and normal skin	25% poor; 20% fair; 25% good; 20% excellent; 10% clear	50% fair or poor outcome, requiring surgical excision
Zeng^18^	Case report	25-year-old female, right eyelid with 1.2 × 1.5 cm in size in the lower eyelid and 0.8 × 1 cm in the upper eyelid	CO2 laser for 15 minutes foreach lid	Normal tissues became visible after ablation; Thermal damage of the lower dermis avoided	Patient satisfied	Edema, incrustation, and erythema; Minimal repigmentation of upper lid; Superficial scar of the lower lid; Hypopigmentation on both wounds at the five-month follow-up
Abrusci and Benzecry^19^	Case report	16-year-old female patient with medium right lower eyelid congenital nevus	CO2 and Er:YAG laser simultaneously	Total reepithelialization within 10 days; No complications during two-year follow-up	Excellent and very satisfactory outcomes with absence of scarring	Satisfactory 3 × 7-mm post-inflammatory lightly hyperpigmented area, not corresponding to melanocytic nevus as per dermatoscopic assessment
Ponomarev et al.^22^	Case report	55-year-old male and 30-year-old female patients with congenital melanocytic nevi on the forehead and lower eyelid	511 and 578 nm dual wavelength copper vapor laser radiations; 55-year-old patient: two sessions one month apart 30-year-old patient: three sessions of one-month interval	Immediate grayish appearance, narrow dark brown covering scab two or three days post-treatment, pink color after seven to 10 days when scab fell off	Three weeks healing time and no recurrences during 24-month follow-up	Slight erythema that resolved
Cho et al.^28^	Case report	15 patients (10 women, five men, 13–73 years old) with congenital divided nevi (small, medium sized) involving eyelash-bearing area	Seven patients: laser therapy; Eight patients: concomitant excision or history of previous excision; One patient: skin graft; Three to six sessions to complete the procedure, and the entire procedure performed every four to six weeks	Assessment by checking postoperative lid margin irregularities, lid notching, delayed wound healing and closure, infections, partial/total decrease in hair along margin and recurrence	Patients’ satisfaction assessment through questionnaire; All patients satisfied	Partial loss of cilia in all patients
McDonnel and Mayou^32^	Case report	Newborn female infant with left 2 × 2.5-cm pigmented divided nevus associated with thickening of the upper and lower eyelids and mild left eye ptosis	Dermabrasion	Decreased nevus size and pigmentation; Size and color reverted to initial presentation after four weeks	Two months postoperative assessment satisfactory with no pigmentation recurrence	None mentioned
Yap and Earley^33^	Case report	4-year-old boy with left congenital periorbital pigmented nevus; 25-year-old woman with congenital pigmented periorbital naevus associated with bleeding after minor trauma; 4-year-old girl with right congenital periorbital pigmented nevus; 23-year-old woman with congenital pigmented periorbital naevus affecting both right eyelids and right infraorbital region	4-year-old boy: nevus excision then reconstruction using full-thickness post-auricular skin graft; 25-year-old woman: lower-lid excision, including eyelashes, and reconstruction using full-thickness post-auricular skin grafts; 4-year-old girl: two-stage excision, reconstruction using full-thickness post-auricular skin graft, wedge excision required two years later; 23-year-old woman: numerous excisions and reconstruction using full-thickness post-auricular skin grafts	4-year-old boy, 25-year-old and 23-year-old women histology: benign compound nevus; 23-year-old woman: further wedge excision necessary two years after original excision; 4-year-old girl histology: intradermal pigmented nevus with extension to conjunctiva	Good or excellent result as judged by patient and/or parents	4-year-old girl: ophthalmologic opinion on scleral and conjunctival melanosis, but grafting not considered; 25-year-old woman: post-operative ectropion, corrected using lateral wedge excision; 23-year-old woman: epiphora due to close excision in lacrimal apparatus region
Papadopoulos et al.^35^	Case report	Four patients (three females, one male, aged from 17 to 54 years old)	Full-thickness excision of one-quarter of the lid and full-thickness skin grafts tocover defects	Full-thickness pentagon excision useful in debulking of nevi extending in posterior lamella of eyelids, maintaining good lobe apposition, and preserving eyelashes line	Successful	None mentioned
Yildirim et al.^34^	Case report	16-year-old boy with continuous pigment lesion affecting upper and lower left eyelid, extending vertically to tarsal conjunctiva of both eyelids	3-4 mm of normal skin surrounding nevus and 1-2 mm from tarsoconjunctival area removed; Newly formed eyelid margin covered by musculocutaneous flap; Minimal lateral tarsorrhaphy to reduce interpalpebral width	Follow-up at six months; Use of lateral musculocutaneous flap for successful results; Patient’s age, gender, diameter of lesion and degree of involvement of tarsus to be kept in mind before surgery	Satisfactory six months post-surgery	None mentioned
Ghosh et al.^36^	Case report	39-year-old female with large kissing nevus spanning central two third of lower and upper left eyelids	Surgical reconstruction in one-stage fashion, by splitting posterior and anterior lamellae and debulking upper/lower lid lesions	Follow-up atone week; Healing of upper lid defect at follow-up, lower lid graft taken nicely with good lid position and no exposure	Great aesthetic and functional results	None mentioned
Rajput et al.^9^	Case report	40-year-old female with melanocytic nevi involving both eyelids	Surgery with canthotomy and cantholysis; Lid repair in both lids by direct suturing method	Histology: stratified squamous epithelium with nests of nevus cells at dermal-epidermal interface	Healed lid skin and improved visual acuity on follow-up	Healing of lid skin; Improvement of visual acuity to 6/36 in left eye
Bayramiçli et al.^37^	Case report	14-year-old boy with congenital panda nevus involving upper and lower eyelids, eyebrow, part of the malar eminence and nasal dorsum	Staged excision of the lesion and defect; Reconstruction with expanded forehead flaps and temporal island flap with hair-bearing skin	After three months: surgery for minor revisions; After 14 months: final stage of surgical treatment	Satisfactory functional and aesthetic results	None mentioned
Desai et al.^38^	Case report	8-year-old male patient with congenital nevocellular nevus	Excision and local advancement flap with V to Y closure of defect lateral to lateral canthus and full-thickness skin graft for eyelid reconstruction	Final pathology: congenital nevocellular nevus	Satisfactory	No complications
Kasai and Ogawa^39^	Case report	16-year-old boy with nevus involving lower and upper eyelids (8 mm inferior and 5 mm superior to eyelashes, respectively)	Surgical excision using the Kuhnt-Szymanowski procedure, and simple excision on the upper eyelid	No ectropion within 11 months follow-up, and nearlyinvisible scar	Complete removal of the lower ciliary border nevus	None mentioned

Source: elaborated by the authors.

## Discussion

Besides the limited studies related to kissing nevus treatment, this review showed that for small, superficial and well-circumscribed lesions laser therapy is an alternative treatment option^18^^,^ 39. The reason behind the use of the laser is to minimize hypertrophic scar as the nevus was superficial and not parallel to Langer’s lines^19^. The CWCO2 abolished most of the nevus, and SPCO2 perfected the ablation. Although these techniques can cause thermal damage and hypertrophic scarring^40^, the low number of passes and appropriate settings decreased those risks. Abolishment of the CO2 thermal damage with Erbium:YAG laser ablation reduces erythema and reepithelialization time span^13^. Few authors described better outcome by early treatment with laser in infancy^20^, but this and other studies could not conclude the same^21^.

To avoid damage, only the nevus site is excised by staged mosaic punching excision, whereas the deeper nevus tissue is removed by laser. Excision is done by full thickness of the dermis and thin superficial fat layer in order to ensure removal of all the dermal elements. The excised skin should be at least 2 mm in diameter in order to avoid wound-healing interruption, and the width of laser excision was less than 3 mm in the eyelash-bearing area. When the treated lesions are epithelialized, we can perform the next laser session, and three to six sessions are required to complete the procedure, and the entire procedure was performed every four to six weeks^28^.

Due to the location of the congenital divided nevus at the ciliary margin, poor aesthetic and functional impairment might occur. The optimal treatment option would be excision of the periorbital region and intervention at the ciliary margin, that includes the eyelash-bearing area. The lid margin bears important functions, like maintaining smooth margin, lubricating the ocular surface and preventing ocular irritation, and the continuity of the lid margin for the shape of orbital fissure^28^.

If cells and lesions are deep in dermis, full-thickness excision followed by repair with full-thickness skin graft or split skin graft is an excellent alternative. Full-thickness skin grafts can be taken from behind the ear^29^^,^ 41 or from the upper eyelid of the defected or normal eye that has been shown to be more successful as compared to regions behind the ear^41^. In case of malignant transformation of the nevus, Ribuffo et al. proposed radical excision and tarsoconjunctival flap reconstruction^41^. The use of scalp flap with staged reconstruction for eyebrow involvement was described for large divided nevus by Dulanto et al.^42^.

Strategical planning for kissing nevus treatment includes dividing the periorbital region into anatomical zones. Excision was staged depending on the clinical requirements and extent of involvement of the nevus. Wedge excisions that are appropriate to the nevus site were done for the reduction of eyelid excess^33^.

Malignant transformation of the eyelid kiss or split nevus has been described very rarely. The reported incidence of congenital melanocytic nevi with malignancy is highly variable, ranging from 2 to 30% depending on the length of follow-up, with an average lifetime of 14%^43^. Findings indicate varying degrees of risk of transformation in nevi depending on the size, location, and type of lesion.

Two studies both emphasized the importance of size in assessing the risk of malignant transformation, with larger CMN being associated with a higher mortality rate in children presenting with melanoma^28^^,^ 33. Other findings also suggested that a pigmented conjunctival mass later in life should be considered malignant melanoma until proven otherwise, as most nevi appear in the first two decades of life^44^. Regardless of size, full-thickness excision is recommended in yet another study as malignant changes can occur in 2–30% of cases^45^.

Lorentzen et al.^46^ carried out the best long-term study that attempted to estimate the incidence of cutaneous melanoma in large CMN. They got their data from the Danish health system. A national registry of 151 patients with large CMN covering over 60 years was used. The medical records and death certificates of those who died were reviewed to determine if death was caused by cutaneous melanoma. All surviving patients in the registry were contacted by questionnaires, and all replied. Individuals mentioning suspicious lesions or symptoms were brought in for assessment. No patient having had melanoma was alive at the time of the survey. It was determined that 4.6% of patients with significant CMN developed cutaneous melanoma. Between registration and follow-up, there were 33 years on average.

The development of kissing nevi arises from melanoblasts or Schwann cells of neuroectodermal origin^43^. While rare, kissing nevi can undergo malignant transformation, leading to melanoma, which is a rare but deadly form of primary skin cancer^32^. Conjunctival and eyelid melanomas are the most aggressive ones. Inherited mutations in the melanocortin 1 receptor (mc1r) gene have been found to increase the risk of cutaneous malignant melanoma^47^. Melanoma can be classified into four categories based on histopathological and clinical criteria: nodular melanoma, superficial spreading, lentigo maligna, and acral lentiginous melanoma. In stages I and II, melanoma thickness and the presence or absence of ulcerations are used to stage the disease due to their prognostic effect. In stage III, there is regional lymph node involvement, and in stage IV, we find metastases^48^. Breslow thickness is a well-known prognostic indicator for cutaneous melanoma, and it was shown that lesions with a histopathological size of ≤ 0.76 mm are associated with a five-year survival rate of 100% as compared to patients with tumors which invaded 1.5 mm or more who had only a 50 to 60% five-year survival rate^49^.

The presence of regional lymph node metastasis is the single and most important prognostic factor for most solid tumors. The presence of ulceration may be another high-risk histologic feature and can predict nodal metastasis. Also, the presence of metastasis and extracapsular extension increases the risk of nodal recurrence^48^.

Some clinicians consider that the risk of malignant deterioration is justification enough to remove all big CMIN^50^^–^ 54. Cosmetic appearance is another important issue that needs to be taken into account. According to the Consensus Conference on Precursors to Malignant Melanoma, “there is not enough information at this time to propose the prophylactic excision of all congenital nevi,” and treatment should be individualized. Lesions should be periodically photographed, measured, and changes documented^55^.

It is important for ophthalmologists not only to perform early biopsy of suspicious lesions when resection and reconstruction are easy, but also to raise awareness so that patients understand the severity of their disease. This allows us to choose the most appropriate treatment that gives the best prognosis.

In summary, the risk of malignant transformation in nevi and other skin lesions should be evaluated based on a variety of factors, and proper surveillance and management should be undertaken to minimize this risk.

Most adverse events, such as ectropion and entropion, depend on the size and location of the nevus. The pre-treatment planning considering nevus location anteriorly or posteriorly is fundamental to avoid these adverse events.

Complications of surgical treatment in the literature include notch formation and cilia loss^35^, pigmentation^56^, and epiphora^33^. A small notch of the upper lid without effect on the lid closure and cilia loss was present in two out of the seven patients who underwent surgery^35^. Dark brownish pigmentation of the sclera is another adverse side effect which was noted in a 10-month follow up post-eyelid fusion surgery^56^.

The literature details a variety of adverse events associated with CO2, Er:YAG and other laser therapies such as incrustation, erythema and edema, hypopigmentation, post-inflammatory hyperpigmented^18^.

It is crucial to consider the depth of the nevus, since the literature showed that one case treated with the YAG laser resulted in clarification and early repigmentation of the lesion two months postoperatively^57^. The residual melanocytes in the reticular dermis cannot be eliminated with single treatment, and this can cause recurrence^58^.

One of the potential and theoretical risks indicated by laser irradiation is malignant transformation and acceleration^7^. A study conducted by Imayama and Ueda reported that clinical and histological findings of malignant neoplasms such as melanoma were not present upon long-term follow-up after laser treatment^59^.

## Conclusion

In conclusion, this review suggests that the effectiveness and satisfaction with various treatments for PCMN depend on the specific treatment method and the individual patient’s case. Overall, the variability in outcomes emphasizes the importance of further research to better understand the most effective and safe approaches for treating PCMN.

## Data Availability

Data sharing is not applicable.
